# Gender, Geographic, and Socioeconomic Representation in Medical Student Journals: A Cross-Sectional Analysis

**DOI:** 10.7759/cureus.12838

**Published:** 2021-01-21

**Authors:** Ibrahim S Al-Busaidi, Kareem Sharif, Ahmad Hassan

**Affiliations:** 1 Family Medicine, University of Otago, Christchurch, NZL; 2 Neuroscience, University of Pennsylvania School of Medicine, Philadelphia, USA; 3 Psychiatry, Yale University School of Medicine, New Haven, USA

**Keywords:** medical education, medical student, medical student journal, peer review, gender bias, gender, journal leadership, gender disparities, socioeconomic disparities, gender representation

## Abstract

Introduction

Women make up the majority of medical school students in most high-income countries. Despite this, women remain underrepresented in senior academic leadership positions including editorial boards of mainstream biomedical journals. Many studies show the underrepresentation of women in mainstream medical journals; however, gender representation in medical student journals (MSJs) is not well documented. Assessing diversity and inclusion in MSJs is vital to understanding the point at which biases in academic medicine are established. Understanding when biases in medical authorship manifest may allow for a more targeted approach to alleviating these biases. This study explores diversity in MSJs by examining gender representation on editorial boards, geographic region, and socioeconomic status of the country of origin.

Methodology

In November 2019, Google^©^, Yahoo!, and Bing search engines as well as PubMed and Google Scholar databases were searched for English-language MSJs using standardized criteria. The websites of identified journals were screened and relevant journal and editorial board-related data were collected. The gender of board members was determined using a sequential approach.

Results

A total of 21 MSJs were included with over half (n = 12, 57.1%) established during the last decade (median years of operation = 9, range = 3-97 years). Most MSJs (n = 17, 81%) are based in North America and Europe. All but one (published in an upper-middle-income country) of the 19 journals originating from a specific country are published in high-income countries. Of the total 348 board members identified (33 editors-in-chief and 315 other editors), 169 were women (48.6%) and 179 were men (51.4%). Women occupied 48.5% of editor-in-chief positions and 48.6% of other editorial board roles.

Conclusions

The gender gap in medical journal leadership appears early during medical education and continues to widen after joining the workforce. Geographic and socioeconomic disparities present in mainstream medical journals also extend to MSJs. Future research should seek to determine whether gender bias is also seen in medical student authorship across MSJs. Approaches to minimizing gender gaps in medical journal leadership should target current medical students as the biases begin to manifest during this period of their education.

## Introduction

Medical students have historically played a significant role in the publication of clinical research and the discovery of landmark findings in medicine. The discovery of insulin, heparin, and the sinoatrial node are just a few of their landmark contributions to medicine [1]. Recently, medical schools have emphasized the importance of early exposure to research by actively incorporating research training into undergraduate medical education [2]. This oftentimes takes the form of compulsory research experience for graduation, research electives, intercalated research degrees, and summer studentships. These research experiences, voluntary or mandatory, can result in tangible output.

Peer review is an exceedingly critical step in the scientific communication process tasked with safeguarding scientific integrity and ensuring the high quality of generated knowledge. However, the long and painstaking process of publishing a manuscript often deters many student researchers from contributing their scholarly work [3]. As such, medical student journals (MSJs)-student-led periodicals that publish student-authored articles-provide a platform with which medical students may be exposed to the process of publishing their work without the many barriers associated with publishing in mainstream journals [4,5]. Feedback provided by reviewers helps medical students improve their research to a publishable caliber while simultaneously acting as a teaching tool for academic writing [4]. Furthermore, a more welcoming environment permits medical students to take research initiatives and become active participants in their fields of interest. Editorial boards of MSJs are led by medical students with supervision and support from faculty advisors [4,5]. Despite concerns related to the transparency and quality of peer-review policies adopted by MSJs and the presumed low quality of published articles, recent evidence suggests that publication in an MSJ is associated with long-term academic achievement [6]. Over 20 MSJs have been established worldwide, and with the increasing emphasis on undergraduate research and other scholarly activities, MSJs are becoming increasingly more relevant [5].

The number of incoming female U.S. medical students has grown consistently over the past decade with 2017 being the first year when female matriculants surpassed males [7]. Additionally, the proportion of female medical students in the U.S. increased from 46.9% in 2015 to over half (50.5%) in 2019 making women the majority of medical students in the U.S. Similar trends in the “feminization of medicine” have also been observed in Europe, Canada, and the Middle East [8,9]. This trend is also seen in graduate-level education at large. For example, women comprised over half of first-time U.S. graduate students at the master’s degree and certificate level (59.7%) and at the doctoral level (54.4%) in 2018 [10]. Additionally, the majority of graduate certificates (64.8%), master’s degrees (58.3%), and doctoral degrees (53.0%) were awarded to women during the academic years 2017-2018. In fact, 2017-2018 marked the eighth consecutive year in which women were awarded the majority of doctoral degrees by U.S. institutions. Despite these trends, women remain underrepresented in senior academic and leadership positions [11,12]. Gender distribution on editorial boards of biomedical journals is commonly utilized to determine the extent of gender bias in academic medicine. Previous studies have found women to be significantly underrepresented in editorial boards of journals in various fields such as anthropology, economics, mathematics, and medicine [12-14]. Additional diversity variables, such as geographical location and country income level of editorial members, are often highly relevant [15].

We previously examined editorial board gender representation in one MSJ over a 14-year period, and while we found no evidence of a gender gap in authorship of original research or editorial board positions, journal issues were significantly more likely to have a male editor-in-chief [16]. No prior research has specifically investigated gender and other biases across MSJs. This study examines diversity in English-language MSJs in terms of gender representation on editorial boards, geographic region, and the socioeconomic status of the country of origin.

## Materials and methods

Search strategy

In the first week of November 2019, we searched Google© (www.google.com), Yahoo! (www.yahoo.com), and Bing (www.bing.com) search engines, as well as PubMed and Google Scholar bibliometric databases, using the term “medical student journal.” The top 300 unique results were manually screened by the first author (ISA) to retrieve the names and websites of MSJs. To avoid confounding the results, we ensured that browsing and search histories were deleted before conducting all searches in a newly downloaded Google Chrome™ on a Windows web browser, version 78.0.3904.87 (Google LLC, Menlo Park, CA, USA). For the purposes of this study, MSJs refer to scientific periodicals that are run and editorially managed, in part or fully, by students [4,5]. We only included currently active MSJs that are published in English with cumulative years in operation of two years or more. This is to allow sufficient time for MSJs to mature editorially after their launch. MSJs that did not include students in their editorial board or did not list the names of editors/board members on their websites were excluded from the study.

Data collection

Two authors (ISA and AH) independently screened the websites of identified MSJs for inclusion as per the eligibility criteria. Any disagreement was resolved by discussion and consensus after the completion of data collection. Websites of identified MSJs were closely examined, and pertinent journal and editorial board data were collected. These included year established, country of origin’s geographic region (by continent) and socioeconomic status, cumulative years in operation, and the publisher. The socioeconomic status of countries was categorized into four groups (low-income, lower-middle-income, upper-middle-income, and high-income economies) based on the World Bank 2020 income classification [17].

Editorial board-related data included names and number of editorial leadership positions (editor-in-chief) and other board members (deputy, section, and associate editors). Information on editorial board members in advisory, oversight, production (e.g., production editors, managing editors, editorial assistants), or supporting roles (e.g., consultants, secretaries, treasurers, and web developers) were not collected.

We determined the gender of board members using a sequential approach, as previously described [15]. First, two authors (ISA, AH) independently inspected the website of each MSJ for information regarding the gender of their board members (e.g., documented gender, photographical evidence). If this was not available or there was no consensus between the authors, then the names of the board members in question were inputted into the validated web application Genderize.io [18], an open source software that assigns a gender to the name using a probabilistic certainty score. A high probabilistic certainty score of ≥0.95 was required for gender assignment to be accepted [15].

Data analysis

Data from identified MSJs were entered into an Excel spreadsheet (Microsoft Corp., Redmond, WA, USA). Descriptive analyses were used to describe the majority of the data. The chi-square goodness-of-fit test was used to determine variance from an equally proportioned distribution for editor gender. Statistical significance was determined if type I error rate was <5% (p-value < 0.05). Analysis of data was undertaken using SPSS for windows version 20.0.0 (IBM, Armonk, NY, USA).

## Results

A total of 23 MSJs were identified. Two MSJs (Amsterdam Medical Student Journal and Student British Medical Journal) were excluded due to incomplete or missing information on editorial board members leaving 21 journals that were included in the study (Table 1). The median years of operation was 9 (mean = 20.5, range = 3-97) with over half (n = 12, 57.1%) established during the last decade (2010-2019). Most MSJs (95.2%, n = 20) are published by academic publishers. Of the included journals, 11 (52.6%) originate from North America, six (28.6%) from Europe, two (9.5%) from Australasia, and two (9.5%) are international MSJs. Of the 19 journals originating from a specific country, 18 are published in high-income countries while one (Turkish Medical Student Journal) is published in Turkey, an upper-middle-income country.

**Table 1:** Medical student journals included in the study. N/A = Not applicable; NZ = New Zealand †Publication of these journals was interrupted. McGill Journal of Medicine was established in 1947 but ceased publication in 1951. It was relaunched in 1994 and ceased again in 2011. It was relaunched for the second time in 2015. University of British Columbia Medical Journal was established in 1962 but ceased publication in 1968. It was relaunched in 2009.

Journal Name	Country	World Bank Income Level	Year established	Cumulative Years in Operation (as of December 2019)
Australian Medical Student Journal	Australia	High	2010	10
American Medical Student Research Journal	USA	High	2013	6
British Student Doctor Journal	Britain	High	2017	3
Cambridge Medicine Journal	Britain	High	1978	41
Dalhousie Medical Journal	Canada	High	1936	41
Florida Medical Student Research Journal	USA	High	2015	4
Harvard Medical Student Review	USA	High	2014	4
International Journal of Medical Students	N/A	NA	2013	7
Journal of the Asian Medical Students’ Association	Asia-Pacific	NA	2012	7
Journal of Medical Students, Galway	Ireland	High	2014	5
McGill Journal of Medicine^†^	Canada	High	1947	25
Medical Student Press Journal	USA	High	2014	5
Medical Student Research Journal	USA	High	2011	9
McMaster University Medical Journal	Canada	High	2003	16
New Zealand Medical Student Journal	NZ	High	2003	16
Royal College of Surgeons in Ireland Student Medical Journal	Ireland	High	2007	12
Turkish Medical Student Journal	Turkey	Upper middle	2014	6
University of British Columbia Medical Journal^†^	Canada	High	1962	17
University College Dublin Medical Student Journal	Ireland	High	2012	8
University of Toronto Medical Journal	Canada	High	1923	97
Yale Journal of Biology and Medicine	USA	High	1928	91

The total number of editorial board members was 348 (median = 14, range = 7-37 per MSJ). Of these, 169 were female (48.6%) and 179 were male (51.4%) (p = 0.59). There were a total of 33 editors-in-chief (median = 1, range = 1-4), 17 of which were male (51.5%) and 16 were female (48.5%) (p = 0.86). Other editorial board members (total = 315, mean = 15, median = 12, range = 6-36) comprised 153 females (48.6%) and 162 males (51.4%) totaling 315 (p = 0.61).

## Discussion

Assessing diversity in MSJs is vital to understanding the point at which gender, geographic, and socioeconomic biases in academic medicine are established. Our study provides new evidence complementing a preliminary analysis of gender representation in a single MSJ from New Zealand [16]. Although the results of this study do not demonstrate a statistically significant difference between male and female representation in MSJ leadership positions, the authors strongly believe that the data nonetheless supports that a slight gender disparity does exist. The statistical analysis of the data assumes that equal gender representation exists among medical students at large. It is important to analyze the results of this study in the greater context of a rapidly growing female cohort of medical students. Females now comprise a majority of medical students not only in the U.S. but also across Europe and the Middle East. No exact data exist in regards to the percentage of female medical students globally, hence the statistical analysis of the data was limited in that the mathematical assumption of equal gender representation among medical students needed to be made. Females represent a majority of medical students in most of the countries that the included MSJs originate from, and the data show that they comprise a minority of MSJ leadership positions. Taking this context into account, the authors are in agreement that a slight gender bias exists in MSJ leadership positions although the data does not demonstrate any significant difference.

While the results of this study suggest that a slight gender bias exists in MSJ leadership positions, the magnitude of the gender gap in MSJs is not as prominent in comparison to mainstream medical journals [12,15]. For example, a recent analysis of 60 major mainstream medical journals found women to be largely underrepresented in leadership positions across specialties; 17.5% of all editorial board members and 16% of editor-in-chief positions were occupied by women [19]. Additionally, although around 59% of obstetrics and gynecology doctors in the U.S. in 2017 were women, a recent study examining editorial boards in women’s health journals [13] found women (42% of board members) to occupy 41% of editor-in-chief positions, 43% of associate editor positions, 30% of deputy editor positions, 38% section editor positions, and 42% of other editor positions. Overall, our data suggest that gender bias in editorial boards starts early during medical school and continues to widen during and after postgraduate education. This is the first study to analyze gender representation among MSJ leadership positions at large, and the evidence that gender bias exists during medical school can prove to be valuable in the wider context of minimizing gender disparities in the medical field. Previously, it was well-established that large gender disparities exist in mainstream medical journal leadership, but it was unknown whether these disparities began during medical school or manifested at a later time point. Because this study provides preliminary evidence that the gender disparity in medical journal leadership begins to manifest during medical school, initiatives that aim to address gender disparities in medicine may benefit from targeting current medical students.

The majority of MSJs included in our study are based in North America and Europe and published in high-income countries, a finding that is consistent with studies of mainstream medical journals. A recent analysis found that only 35% of editors in 12 specialty global health journals were based in low- or middle-income countries (LMICs) [20]. Another study reported that the vast majority of overall editors (68%) and editors-in-chief (73%) of global health journals were based in high-income countries [15].

While geographic and socioeconomic disparities in MSJs observed in this study are likely caused by a lack of resources in medical schools in LMICs, it may also be that mainstream journals have replaced the need for MSJs in these countries. Perhaps national mainstream medical journals have included avenues for medical students and trainees to publish their research. In the absence of the friendly and supportive peer-review processes afforded by MSJs, medical students in LMICs may be deterred from publishing their scholarly work due to the many barriers to publication in mainstream journals [4,5]. As a result, students interested in research may be discouraged from pursuing academic medicine. Medical schools in LMICs are, therefore, encouraged to establish MSJs to provide avenues for their students to publish their research and gain valuable publishing experience. Meanwhile, further research is required to explore barriers to and facilitators of establishing and maintaining MSJs in LMICs.

Limitations

This study needs to be considered in light of its strengths and limitations. It examined diversity in MSJs across several dimensions including gender representation on editorial boards as well as geographic region and socioeconomic status of the country of origin. In addition, the study utilized a sequential method to confidently determine the gender of editorial board members. Our study is primarily limited by its cross-sectional design which did not permit examining trends in gender and other biases. Additionally, our findings may not be generalizable to all MSJs. First, we only examined journals published in English. Second, some journals may have been inadvertently omitted despite utilizing comprehensive standardized search criteria. Therefore, future studies ought to explore trends in gender and other biases in English- and non-English-language MSJs over the years.

## Conclusions

Our findings suggest that gender disparities in editorial boards of biomedical journals possibly manifest during medical school and continue to widen during postgraduate education and beyond. Geographic and socioeconomic disparities present in mainstream journals also extend to MSJs. Initiatives that seek to address and minimize these disparities should be targeted towards medical students. Previously, it was known that mainstream academic journals exhibit a high degree of gender bias, and these findings now suggest that addressing the importance of gender representation to medical students and MSJ editorial boards may help to decrease the gender gap in medical journal leadership. Additionally, it is important to identify which barriers are causing this gender disparity in MSJs and mainstream academic journals. Addressing these barriers may help to increase the presence of women on editorial boards of MSJs and mainstream journals. Future research should seek to determine whether gender bias is also seen in medical student authorship across MSJs.
